# Volitional and forced running ability in mice lacking intact primary motor cortex

**DOI:** 10.3389/fncir.2025.1630932

**Published:** 2025-08-14

**Authors:** Ryusei Abo, Mei Ishikawa, Rio Shinohara, Takayuki Michikawa, Itaru Imayoshi

**Affiliations:** ^1^Laboratory of Brain Development and Regeneration, Graduate School of Biostudies, Kyoto University, Kyoto, Japan; ^2^Laboratory of Optical Biomedical Science, Institute for Life and Medical Sciences, Kyoto University, Kyoto, Japan; ^3^Biotechnological Optics Research Team, RIKEN Center for Advanced Photonics, Saitama, Japan; ^4^Center for Living Systems Information Science, Graduate School of Biostudies, Kyoto University, Kyoto, Japan; ^5^Laboratory of Deconstruction of Stem Cells, Institute for Life and Medical Sciences, Kyoto University, Kyoto, Japan

**Keywords:** motor control, cerebrum, running wheel, treadmill, cortical injury

## Abstract

The coordination of various brain regions achieves both volitional and forced motor control, but the role of the primary motor cortex in proficient running motor control remains unclear. This study trained mice to run at high performance (>10,000 rotations per day or >2,700 rotations per hour) using a running wheel, and then assessed the effects of the removal of bilateral cortical areas including the primary motor cortex on volitional (self-initiated) and forced (externally driven) running locomotion. The control sham-operated group revealed a quick recovery of volitional running, reaching half of the maximum daily rotation in 3.9 ± 2.6 days (*n* = 10). In contrast, the cortical injury group took a significantly longer period (7.0 ± 3.3 days, *n* = 15, *p* < 0.05) to reach half of the maximum volitional daily rotation, but recovered to preoperative levels in about two weeks. Furthermore, even 3 days after surgery to remove cortical regions, the running time on a treadmill moving at 35.3 cm/s, which is difficult for naïve mice to run on, was not significantly different from that in the sham-operated group. These results suggest that the intact primary motor cortex is not necessarily required to execute trained fast-running locomotion, but rather contributes to the spontaneity of running in mice.

## Introduction

Even seemingly simple behaviors like reaching, grasping or walking require a high degree of coordination between various brain regions. Although the rhythms of walking are thought to be produced by a neural circuit in the spinal cord called the central pattern generator (CPG) (Brown, 1911; Grillner and El Manira, 2020), the role of the cerebral motor cortex in the gait control has been the subject of various studies. Canines and felines with cooled motor cortex could walk on flat surfaces but have difficulty walking on ladders and wire mesh, suggesting that the motor cortex is involved in the coordination and adaptation of skilled walking movements rather than in basic gait rhythm generation (Armstrong, 1986). Feline motor cortex neurons exhibit activity changes linked to gait phase (Beloozerova and Sirota, 1993), and electrical stimulation of motor cortex neurons can alter hindlimb movements during walking (Bretzner and Drew, 2005). In mice, spontaneous walking and grooming movements have been reported immediately after damage to the bilateral cortical primary motor cortex (Nicholas and Yttri, 2024). Unilateral ischemic strokes in the motor cortex of the mouse resulted in slight and transitory deficits in the skilled placement of the contralateral fore paw and hind paw without affecting fundamental aspects of locomotion during swimming, walking and wading (Zorner et al., 2010). After traumatic injury to the unilateral mouse sensorimotor cortex, forced walking or running movement on a treadmill appeared normal, and detailed kinetic analysis has only reported minute effects (Ueno and Yamashita, 2011). These reports have in common that the brain mechanisms of gait motor control depend to a small extent on the cortical motor cortex.

When humans and animals move voluntarily at a time of their own choosing, rather than being forced to move, readiness potentials, a slow buildup of electrical potential recorded by electroencephalography, are generated in a specific region of the brain prior to movement (Libet et al., 1983; Schurger et al., 2021). The readiness potential is commonly characterized as having an early and a late component (Libet et al., 1983). The early component (~1,500–400 ms prior to movement onset) is a gradual increase in negativity evoked in the supplementary motor area and premotor cortex, whereas the late component (~400–0 ms) is generated by activity in the primary motor cortex. The readiness potential has traditionally been interpreted as a sign of planning and preparation for movement, but this view is actively debated (Schurger et al., 2021; Triggiani et al., 2023). It is hypothesized that motor volition is generated in the medial prefrontal cortex (Brass and Haggard, 2008; Passingham et al., 2010) and that all voluntary physical movement instructions go through the final stage of the primary motor cortex (Haggard, 2009). However, the difference in the role of the primary motor cortex in executing volitional (self-initiated) and forced (externally driven) running locomotion remains elusive.

Spinning wheel locomotion has been observed in the wild and reported to be part of play, escape and exploratory behavior (Meijer and Robbers, 2014). Spontaneous wheel running is less stressful and closer to mice’s natural locomotor pattern than forced treadmill exercise (Manzanares et al., 2018). Furthermore, running wheels have been used as a model for exercise training, as the number and frequency of wheel rotations can be electronically recorded and analyzed for individual data (Goh and Ladiges, 2015). On the other hand, treadmill testing does not rely on voluntary activity and can be completed in a short time, making it suitable for elucidating the mechanisms involved in motor adaptation and qualitatively assessing walking movements (Dougherty et al., 2016; Schmitt et al., 2020).

**Figure 1:**
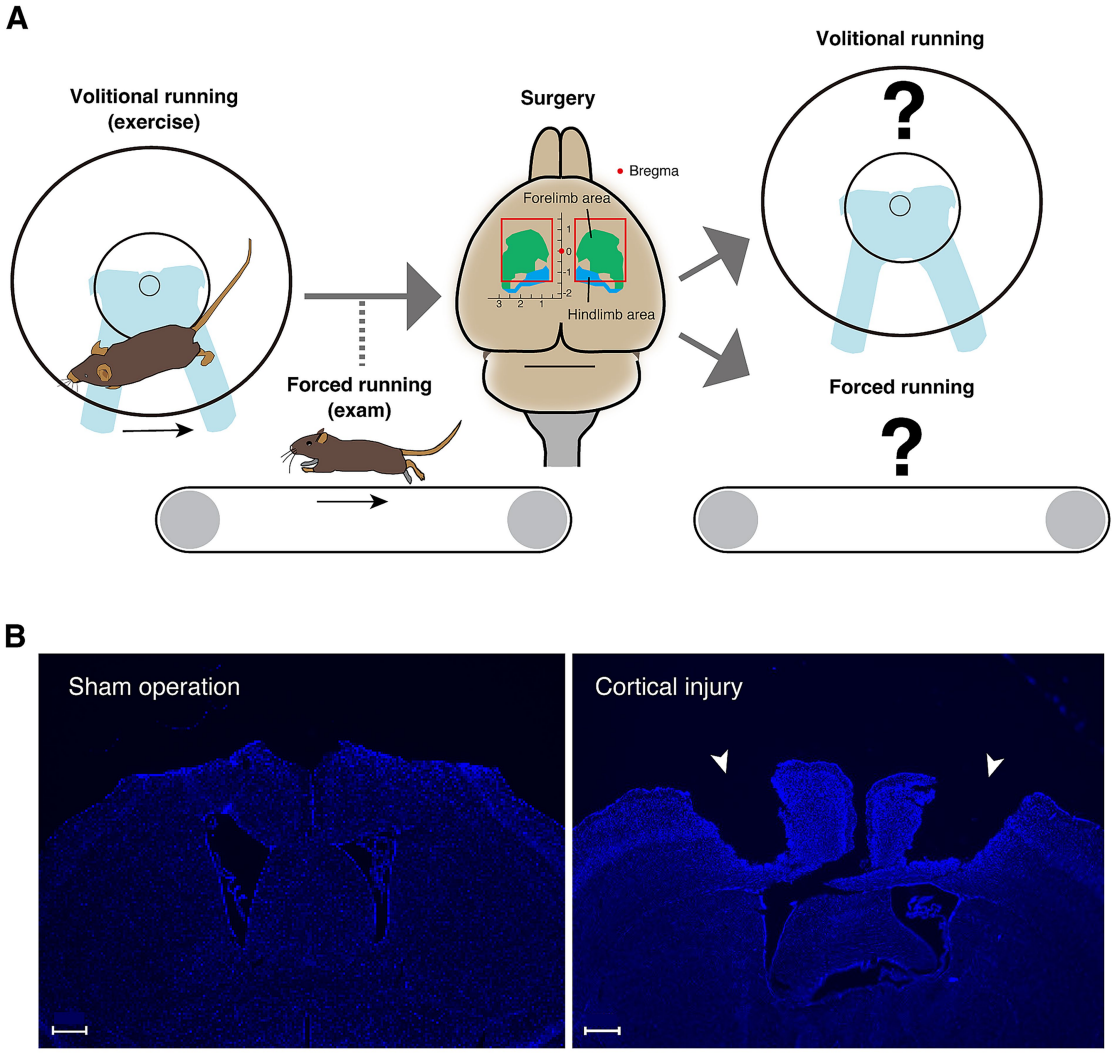
Conceptual diagram of the research strategy. **(A)** A running wheel is introduced in the home cage to encourage mice to perform volitional running movements; once the total number of rotations per day has reached the criteria, running ability is measured on a treadmill. For mice that have acquired sufficient running ability, surgery is performed to aspirate the bilateral cortical areas including primary motor cortex. Volitional running with a running wheel and forced running on a treadmill are then measured. **(B)** DAPI staining images of the cerebral cortical section (0.38 mm from the bregma) of the mouse two weeks after sham operation in the left panel and the section (−0.34 mm from the bregma) of the mouse two weeks after injury in the right panel. Injured sites are indicated by arrowheads. Scale bars: 500 μm.

In this study, mice were trained using a running wheel to acquire the locomotor functions that enable high-speed running. Then, the effects of cortical damage on both volitional and forced high-speed running ability were assessed using a running wheel and treadmill, respectively (Figure 1A). The results elucidated a novel aspect of the role of the primary motor cortex in trained gait motor control.

## Materials and methods

### Animals

Twenty nine wild-type C57BL/6 J mice (CLEA Japan Inc. and Japan SLC Inc.) at 12–15 weeks of age were subjected to cortical injury surgery, in which the neocortical primary motor cortex was removed by aspiration. Eleven mice were subjected to sham surgery, in which the skull was detached and then put back together without cortical injury. Fifteen mice that survived after cortical injury (8 males and 7 females) and 10 mice that survived after sham surgery (3 males and 7 females) were used in the running wheel experiments. Ten cortical injury mice (6 males and 4 females) and 7 sham-operated mice (3 males and 4 females) were used in the treadmill experiments. Animals were randomly assigned to injury vs. sham operation. A flow chart of running wheel and treadmill experiments is shown in Supplementary Figure 1.

Mice were delivered to the breeding racks in the behavioral laboratory building one week prior to the experiment. Individuals were identified by ear holes. Mice were monitored for health and body weight and were housed in Innocage (W234 × D373 × H140 mm) (Innovive) in a temperature- and humidity-controlled room with a 12-h light–dark cycle reversed (dark period from 8 am to 8 pm, light period from 8 pm to 8 am the following morning). The cages were covered with floor matting and fed pelleted food and water in bottles ad libitum. The treadmill forced running experiment was conducted during the dark period, and intervention on the mice by non-test subjects was avoided as much as possible during the experiment.

### Surgery

After 12- to 15-week-old mice were anesthetized with a triad of anesthetics (medetomidine hydrochloride: 0.0225 mg/mouse, midazolam: 0.12 mg/mouse, butorphanol tartrate 0.15 mg/mouse), the scalp was cut along the midline and the primary motor cortex (anteroposterior axis direction with bregma as origin: −1.25 ~ +1.50 mm, left–right axis: 0.50 ~ 2.75 mm, depth: 0 ~ 1.00 mm) over the cranium, which was removed with an electric drill to remove bone and the dura mater was stripped to expose tissue. Exposed cortical tissue was removed using an aspirator fitted with a 1 mm diameter 200 μL tip until the tissue was light in color and close to the corpus callosum. The aspirated primary motor cortical area was filled with Spongel (LTL Pharma) moistened with saline to stop bleeding and the removed bone was returned. This procedure was performed bilaterally. The open scalp of the mice was then sutured and the three anesthetic antagonists atipamezole (0.0225 mg/mouse), the antibiotic carprofen (0.2 mg/mouse) and dexamethasone (0.4 mg/mouse) were administered intraperitoneally.

A control group was also prepared in which only tissue was exposed without injury and then sutured (sham-operated group). In the sham-operated group, the exposed brain surface was kept moist with saline so that the operation time was similar to that of the cortical injury group, and the skull was returned and the scalp sutured after being left for a time equivalent to the 15 min for the suction operation in the cortical aspiration group.

### Measurement of volitional running locomotion using a running wheel

Battery-powered wheel rotation counter were constructed as described previously (Terstege and Epp, 2024). An electrical circuit using a microcontroller board (Arduino UNO R3), a magnetic sensor KY-003 and a real-time clock module DS3231 (Terstege and Epp, 2024) was used to record wheel rotation time. To record total daily movements in the home cage, the rechargeable AA batteries were changed once a day, and the mice’s runs were recorded continuously day after day. Note that the recording to the SD card is often incomplete and not every day a recording was made for each individual mouse. A running wheel (LIFKOME, 18 cm diameter) was introduced into the mouse cage daily from 12 to 19 days before surgery. To avoid the influence of light on the diurnal rhythm, battery replacement and data acquisition were carried out in the dark during the dark period. The number of rotations was measured for 24 or 22 consecutive hours per day.

### Measurement of forced running ability using a treadmill

The experiment using the treadmill was based on the method of Dougherty et al. (2016) and was performed as follows (Supplementary Figure 2A). This experiment was performed during the dark periods of the day before, 3 days, 1 week and 4 weeks after surgery. The treadmill (MISUMI, SVKR-150-400-6NV-NH-NM-H-R, Supplementary Figures 2B–D) was darkened by placing a ceiling at the end of the running direction, and the rest of the treadmill was covered with transparent acrylic panels to prevent jumping out. The mice were placed on a treadmill that did not move for the first 60 s. Over the next 60 s, the mice were allowed to run while increasing their speed from 4.4 cm/s to 17.7 cm/s, and then on the treadmill moving at 17.7 cm/s for the next 60 s. The running direction was instantly switched and the mice were allowed to run at 17.7 cm/s for 60 s in the same manner. The speed was increased from 17.7 cm/s to 35.3 cm/s in 200 s and finally 35.3 cm/s for 300 s. If the mice were unable to run midway, the treadmill was stopped and the mice were retrieved. The upper half of the direction of travel of the mice on the treadmill was covered with a top plate. Under conditions requiring a high speed of 35.3 cm/s and sufficient running ability of the mice, the mice predominantly traveled in a dark area (Supplementary Figure 2C). On the other hand, when the mouse is temporarily stationary, the mouse appears in a bright area without a top plate (Supplementary Figure 2D). If this condition persists and running becomes impossible, the mouse hits the wall behind it. In order to quantify the running function of the mouse, the total time the mouse was hidden by the top plate when the treadmill was driven at 35.3 cm/s was measured.

### Fluorescence staining

Mice were deeply anesthetized with a triad of anesthetics followed by perfusion fixation in phosphate-buffered saline (PBS) and 4% paraformaldehyde (PFA)/PBS (pH 7.4). Brains were fixed overnight at 4°C in 4% PFA and replaced every 24 h with 10, 20% or 30% sucrose/PBS. After replacement was completed, brains were embedded in an embedding agent (OCT compound, Tissue-Tek) for frozen tissue section preparation and frozen at −80°C. Brain sections (50 μm thick) were prepared in a cryostat (Leica CM1950).

For fluorescence staining, frozen sections were incubated with 5% normal donkey serum and 0.1% Triton X-100/PBS for 1 h at room temperature for blocking. Afterwards, the sections were washed with PBS and stained with DAPI (4′,6-diamidino-2-phenylindole, Invitrogen, #D21490) (dilution ratio: 1:5000). Sections were attached to glass slides and photographed under a fluorescence microscope (KEYENCE, BZ-X800) (objective: ×2, NA = 0.1, #BZ-PA02; filter: BZ-X filter DAPI, excitation: 360/40 nm; emission: 460/50 nm, #OP-87762).

### Data analysis

To determine the extent of recovery of volitional running locomotion after surgery, the maximum number of rotations per day in each mouse was determined by fitting a sigmoid function to the total number of rotations per day and the number of days it takes to reach half of that value was calculated. The sigm_fit function (https://jp.mathworks.com/matlabcentral/fileexchange/42641-sigm_fit) was used to fit the sigmoid function. The goodness of fit was determined by the following coefficient of determination *R^2^*,


$$R^{2}=1-\frac{SS_{\mathrm{res}}}{SS_{\mathrm{tot}}}$$

$$SS_{\mathrm{res}}=\sum_{i}(y_{i}-f_{i})$$

$$SS_{\mathrm{tot}}=\sum_{i}{\left(y_{i}-\bar{y}\right)}^{2},$$

where, *y_i_* is the *i*-th observed value and *f_i_* is its associated predicted value, *SS_res_* is the residual sum of squares (difference between the observed and predicted values), and *SS_tot_* is the total sum of squares (difference between observed values and their mean $\bar{y}$). *R^2^* values for sigmoid fitting of all running wheel data were 0.580 ± 0.258 (mean ± standard deviation, *n* = 55). Student’s t-test was used to compare the number of days it takes to reach half of the maximum wheel rotations between the sham-operated and cortical injury groups. Statistical analysis of the maximum number of rotations per day in volitional wheel running was performed using the multiple comparison method with the Tukey–Kramer method with the multicompare function in the Statistics and Machine Learning Toolbox of MATLAB, followed by a one-factor analysis of variance (ANOVA). MATLAB function *kstest* in the Statistics and Machine Learning Toolbox was used to test the normality of distributions. If the *p* value was greater than 0.05, the distribution was considered not significantly different from the normal distribution. For the treadmill data analysis, the time that mice were in the dark was measured using a custom-made MATLAB code and multiple comparisons were performed using the Tukey–Kramer method. MATLAB function *ncfcdf* in the Statistics and Machine Learning Toolbox was used to calculate statistical power at unequal sample sizes or required sample size with a significance level of 0.05.

## Results

### Cortical injury surgery

Based on the structure of the motor cortex of C57BL/6 mice, which was determined from the measurement of movements evoked by intracortical micro-stimulation and the histological structure of the cortex (Tennant et al., 2011), the motor cortex including the forelimb and hindlimb areas was removed by aspiration (the anteroposterior axis direction: −1.25 ~ +1.50 mm, the left–right axis direction: 0.50 ~ 2.75 mm, and the depth: 0 ~ 1.00 mm with bregma as the origin) (Figure 1A). As a control group, a ‘sham operation group’ was created in which the same procedure was performed up to skull and dura mater removal, and the skull was returned and scalp sutured without aspiration of the cortex. Mouse brain sections with sham operation and cortical injury surgery stained by DAPI are shown in Figure 1B and Supplementary Figure 3.

### Effects of cortical damage on volitional locomotion

**Figure 2:**
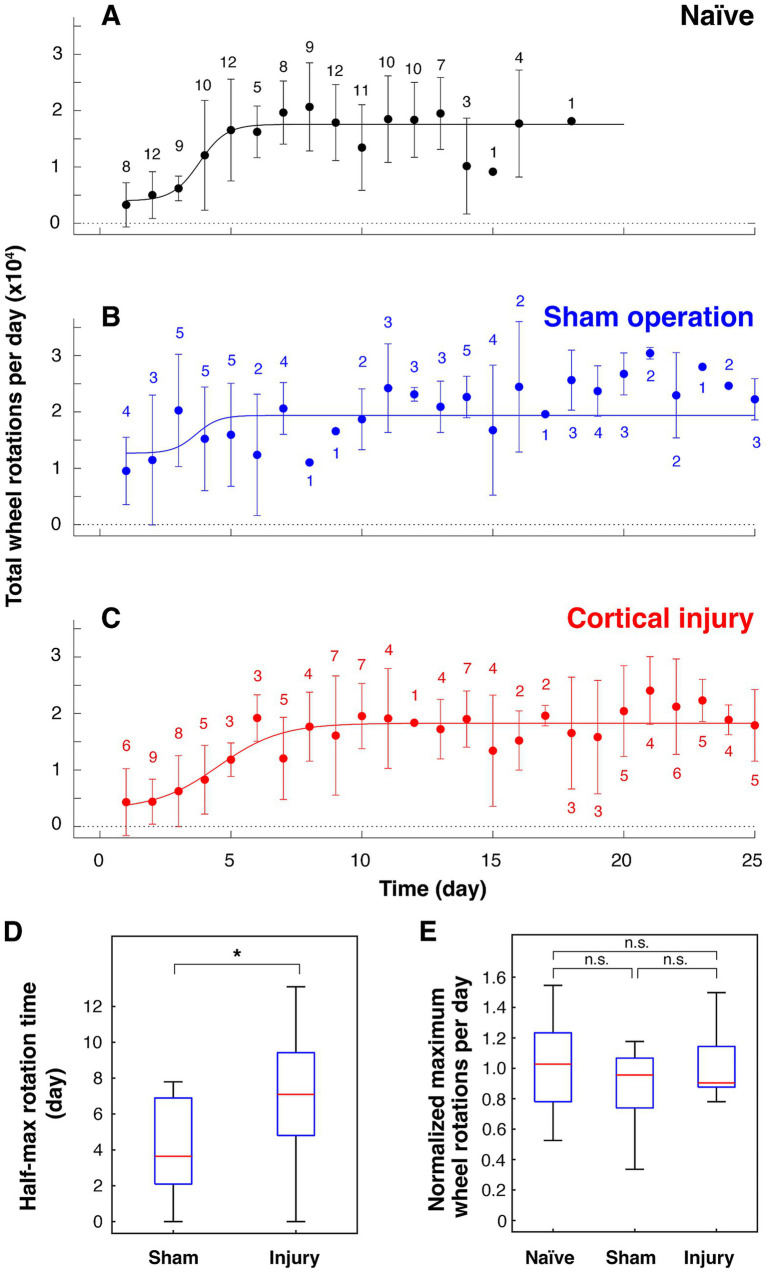
Effects of cortical damage on volitional locomotion in mice. Total wheel rotations per day before and after surgery plotted against elapsed days (mean ± standard deviation). **(A)** Before surgery (16 mice). **(B)** Sham operation group (6 mice). **(C)** Cortical injury group (10 mice). The number of data for each point is noted on the plots. **(D)** Number of days taken to reach half of maximum wheel rotations of sham surgery group (*n* = 10; *p* = 0.879 for rejection of normality) and cortical injury group (*n* = 15; *p* = 0.989 for rejection of normality). * *p* = 0.0226 (Student’s *t*-test, statistical power: 0.729). **(E)** Normalized maximum number of rotations per day against mean of maximum number of rotations per day of naïve mice [22,801 ± 6,065 per 24 h (*n* = 16) and 16,234 ± 4,981 per 22 h (*n* = 14)]. n. s.: not significant (*p* = 0.602, 0.842, and 0.903 for naïve vs. sham, sham vs. injury, and naïve vs. injury, respectively, Tukey–Kramer’s multiple comparison). *p* = 0.867, 0.645, and 0.354 for rejection of normality of naïve (*n* = 30), sham (*n* = 10) and injury (*n* = 15), respectively. When *f* value (0.582) obtained based on the difference between the means of the three groups is used as an effect size with a significance level of 0.05 and a statistical power of 0.8, the total number of samples required is 55, which is met by the results. The results of 24-h measurements are shown in **A–C**, and the results of both 22- and 24-h measurements are shown in **D and E** (see Supplementary Figure 1).

To investigate the effects of cortical damage, the amount of volitional locomotion of mice in the home cage was measured using a running wheel capable of recording the number of rotations. The total number of wheel rotations per day of naïve mice, i.e., mice with no prior running-wheel experience, is shown in Figure 2A. Naïve mice aged 12 ~ 13 weeks took 3.8 days to reach half of the maximum number of rotations per day and about a week to reach the maximum (Figure 2A). Mice with a plateau in running performance (> 10,000 rotations per day and/or > 2,700 rotations per hour) underwent cortical injury surgery or sham operation. The sham-operated group, in which the scalp was sutured without aspirating the cortex after craniotomy, showed a quick recovery, reaching half of the maximum daily rotation in 3.9 ± 2.6 days (*n* = 10) (Figures 2B,D, and Supplementary Figure 4). In contrast, the cortical injury group took 7.0 ± 3.3 days (*n* = 15) to reach half of the maximum daily rotation, which is significantly longer than those of the sham-operated group (Figures 2C,D, and Supplementary Figure 4). However, both groups recovered their maximal daily wheel rotations to preoperative levels (Figure 2E).

### Effects of cortical damage on forced running ability

The effect of cortical damage on running motor function was investigated in an experiment in which the subjects were forced to perform running exercise on a rotating treadmill. The details of the experimental procedures for the treadmill running task are described in the Experimental Methods section.

**Figure 3:**
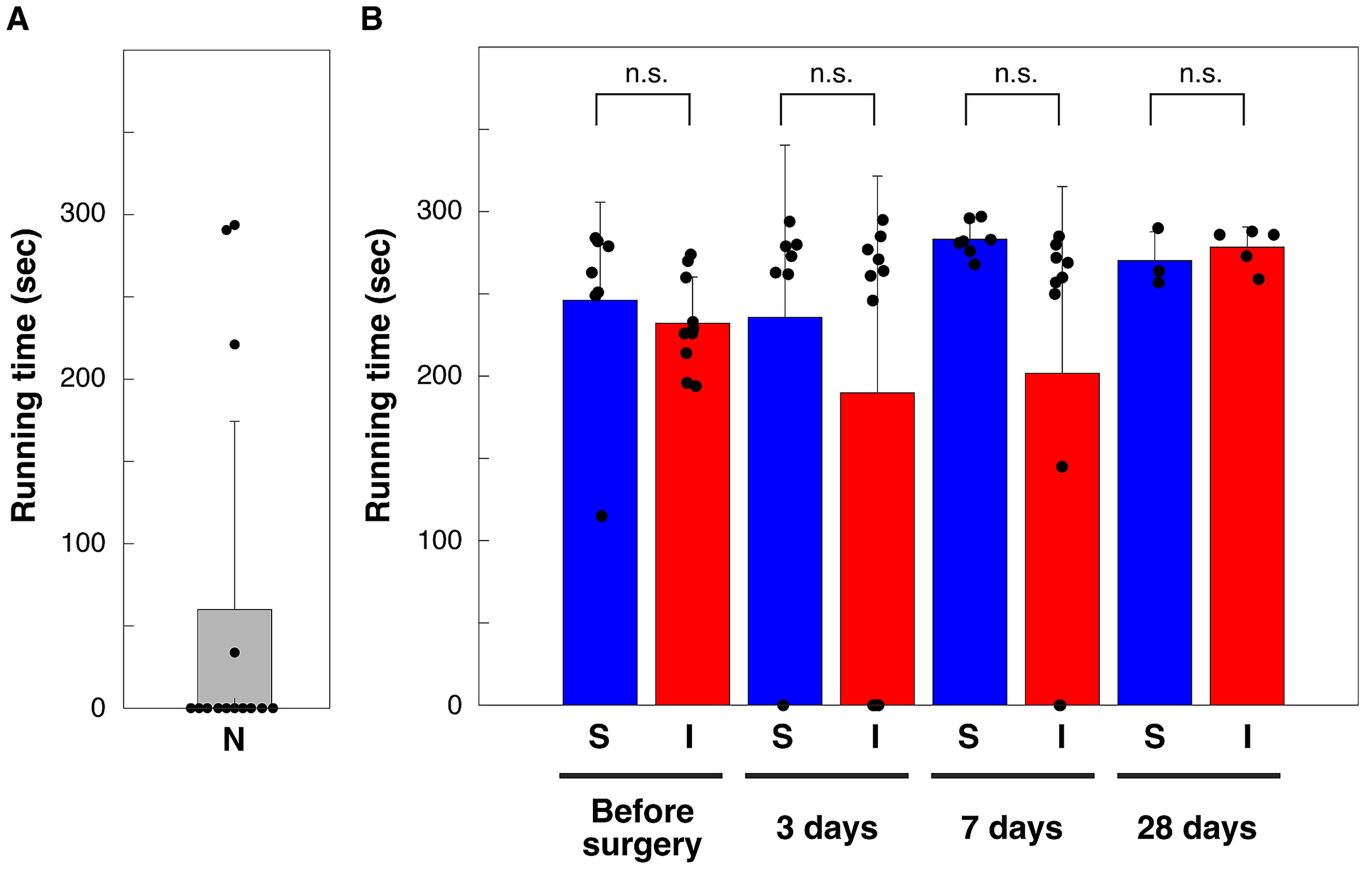
Running time on a treadmill moving at 35.3 cm/s. **(A)** N, naïve mice without running wheel exercise (*n* = 14). **(B)** S, shame-operation group, I, cortical injury group. **p < 0.01; n. s., not significant (*p* = 1.000, 0.954, 0.527, and 1.000 for before, 3 days, 7 days and 28 days after sham-operation vs. cortical injury, respectively), Tukey–Kramer’s multiple comparison. *p* = 0.213, 0.076, 0.800, and 0.810 for rejection of normality of before (*n* = 7), 3 days (*n* = 7), 7 days (*n* = 7), and 28 days (*n* = 3) after sham-operation, respectively. *p* = 0.806, 0.106, 0.107, and 0.544 for rejection of normality of before (*n* = 10), 3 days (*n* = 10), 7 days (*n* = 10), and 28 days (*n* = 5) after cortical injury, respectively. Bars are means, error bars are standard deviations. Individual values are indicated by black circles.

Nine of the 14 naïve mice could not run at all on the 35.3 cm/s treadmill (Figure 3A). Mice trained on a running wheel and achieving running ability beyond the certain standard (> 10,000 rotations per day and/or > 2,700 rotations per hour) were all able to run for more than 180 s out of 300 s on a treadmill moving at 35.3 cm/s (Figure 3B). In this study, these high-performing mice were subjected to cortical injury surgery or sham operation. The running times of these mice when the treadmill was driven at a speed of 35.3 cm/s were compared at preoperative, 3 days, 1 week and 4 weeks after surgery (Figure 3B). Comparison of running times showed no significant differences between all groups between the cortical injury group and the sham operation group (multiple comparison method using the Tukey–Kramer method) (Figure 3B).

## Discussion

### The role of the primary motor cortex on the execution of trained running

The maximum number of rotations per hour for the mice used was approximately 3,000 when wheel rotation was monitored using the method described previously (Terstege and Epp, 2024). This ability to run sustained over an hour or more can only be reached by training for a week (Figure 2A). In the present study, mice that were once able to perform running locomotion at this level were operated on by removing the bilateral cerebral cortex including the primary motor cortex, and the effect on running locomotion ability was investigated. It was found that the sham-operated group remained high in wheel rotations per day after surgery and thus recovered quickly to preoperative levels. In contrast, in the cortical injury group, the number of wheel rotations per day dropped to pre-training levels after surgery and the recovery to the pre-surgery level took about 10 days. These volitional running performance measurements showed that the period of reduced running performance after surgery was longer in the cortical injury group than in the sham-operated group. However, locomotion measurements using a running wheel may be influenced by factors other than the ability to perform locomotion, such as motivation to exercise. We therefore measured forced running ability on a treadmill moving at a speed of 35.3 cm/s, which is difficult for naïve mice to run on. The results showed that there was no significant difference in the time they could run compared to mice in the sham-operated group, even 3 days after cortical injury. These results suggest that the ability to run at high speeds was not compromised by cortical damage. In other words, the intact primary motor cortex is not necessarily required for the execution of trained running locomotion in mice, which is consistent with previous studies (Armstrong, 1986; Zorner et al., 2010; Ueno and Yamashita, 2011; Nicholas and Yttri, 2024). It has been shown that motor learning involves synaptic plasticity in pyramidal cells of the primary motor cortex (Fu et al., 2012; Xu et al., 2009; Yang et al., 2009). There may be a mechanism by which memories related to motor learning are transferred to other brain regions outside the primary motor cortex during gait motor mastery.

### Neural mechanism underlying gait initiation and cessation

The mesencephalic locomotor region (MLR) (Orlovsky et al., 1999) is located in and near the mesencephalic and pedipalp bridge capsular nuclei where continuous micro-electrical stimulation induces walking movements on a treadmill. The MLR, which projects to the nucleus reticularis gigantocellularis and nucleus reticularis magnocellularis and contributes to the activation of the spinal gait CPG. The basal ganglia constantly inhibits the MLR and the disinhibition initiates walking movements (Roseberry et al., 2016). As the basal ganglia receive excitatory input from the cerebral cortex (Arber and Costa, 2022; Gomez-Ocadiz and Silberberg, 2023; Shepherd, 2013), the cortex controls basal ganglia activity is thought to be involved in the initiation and cessation of gait locomotion. However, so far it is not clear which areas of cortical activity are involved in the initiation and cessation of walking movements. The results of this study suggest that the primary motor cortex may contribute to volitional gait initiation. This is because it took significantly longer for the cortical injury group to reach half of their daily maximum volitional rotation than the sham-operated group, even though the former’s forced running ability was indistinguishable from that of the latter. However, the injured group eventually recovered to preoperative levels of maximum daily wheel rotations, suggesting that there may be neural circuits that can initiate movement even when the intact primary motor cortex is missing. Further investigations of the relationship between activity in areas other than the primary motor cortex, such as the posterior parietal cortex (Drew and Marigold, 2015), and walking movements will help to elucidate the brain mechanisms that control the timing of gait initiation and cessation.

## Conclusion

In this study, mice were trained to run at high speed on a running wheel to evaluate the effects of removal of bilateral cortical areas, including the primary motor cortex, on both volitional running on a running wheel and forced running on a treadmill. Results showed that the primary motor cortex was not necessarily required for trained high-speed running, but contributed to the spontaneity of running. This new aspect of the role of primary motor cortex in trained running movements will help to elucidate gait motor control and will make a clinical contribution to the rehabilitation of stroke and cortical injury.

### Limitations of the study

As shown in Figure 3B, forced locomotor performance on the treadmill at 3, 7, and 28 days postoperatively was not significantly different between the sham operation and cortical injury groups. However, a post-hoc power analysis indicates that a sample size of 6 ~ 11 animals per group is required to meet a significance level of 0.05 and a statistical power of 0.8. Thus, the possibility remains that the effect of cortical damage on forced running was not detected. We also did not examine the effect of gender on the effect of cortical damage. The analysis may include a ceiling effect because mice were selected for their ability to run on a running wheel before surgery.

## Data Availability

The raw data supporting the conclusions of this article will be made available by the authors, without undue reservation.

## Supplementary material

Supplementary Figure S1 Flowchart of the experiments with running wheels and treadmill. M: male, F: female.

Supplementary Figure S2 Forced running experiment on a treadmill. **(A)** Speed of treadmill rotation. **(B)** Treadmill experimental apparatus. **(C)** Video image during running. **(D)** Video image of mice unable to keep up with the speed of the treadmill.

Supplementary Figure S3 DAPI staining images. The cerebral cortical sections of mice (1_1, 5_1, 7_1, 8_1, and 10_1) after 2, 3, 1, 4 and 4 weeks after cortical injury surgery, respectively, in the left, and the sections of mice (6_1, and 7_7) 2 weeks after sham-operation in the right. Injured sites are indicated by arrowheads. Scale bars: 500 μm.

Supplementary Figure S4 Effects of cortical damage on volitional locomotion. Total wheel rotations per day before and after surgery plotted against elapsed days (mean ± standard deviation). The data shown in Figures 2A–C are superimposed on a single figure.

## References

[ref1] Abo R. Ishikawa M. Shinohara R. Michikawa T. Imayoshi I. (2025). Volitional and forced running ability in mice lacking intact primary motor cortex. bioRxiv. doi: 10.1101/2025.05.14.653913 PMC12391011 40896520

[ref2] Arber S. Costa R. M. (2022). Networking brainstem and basal ganglia circuits for movement. Nat. Rev. Neurosci. 23, 342–360. doi: 10.1038/s41583-022-00581-w, PMID: 35422525

[ref3] Armstrong D. M. (1986). Supraspinal contributions to the initiation and control of locomotion in the cat. Prog. Neurobiol. 26, 273–361. doi: 10.1016/0301-0082(86)90021-3, PMID: 3526411

[ref4] Beloozerova I. N. Sirota M. G. (1993). The role of the motor cortex in the control of vigour of locomotor movements in the cat. J. Physiol. 461, 27–46. doi: 10.1113/jphysiol.1993.sp019499, PMID: 8350266 PMC1175243

[ref5] Brass M. Haggard P. (2008). The what, when, whether model of intentional action. Neuroscientist 14, 319–325. doi: 10.1177/1073858408317417, PMID: 18660462

[ref6] Bretzner F. Drew T. (2005). Contribution of the motor cortex to the structure and the timing of hindlimb locomotion in the cat: a microstimulation study. J. Neurophysiol. 94, 657–672. doi: 10.1152/jn.01245.2004 15788518

[ref7] Brown T. G. (1911). The intrinsic factor in the act of progression in the mammal. Proc Roy Soc Lond Ser B 84, 308–319.

[ref8] Dougherty J. P. Springer D. A. Gershengorn M. C. (2016). The treadmill fatigue test: a simple, high-throughput assay of fatigue-like behavior for the mouse. J. Vis. Exp. 111:54052. doi: 10.3791/54052 PMC4927751 27286034

[ref9] Drew T. Marigold D. S. (2015). Taking the next step: cortical contributions to the control of locomotion. Curr. Opin. Neurobiol. 33, 25–33. doi: 10.1016/j.conb.2015.01.011 25643847

[ref10] Fu M. Yu X. Lu J. Zuo Y. (2012). Repetitive motor learning induces coordinated formation of clustered dendritic spines in vivo. Nature 483, 92–95. doi: 10.1038/nature10844, PMID: 22343892 PMC3292711

[ref11] Goh J. Ladiges W. (2015). Voluntary wheel running in mice. Curr Protoc Mouse Biol 5, 283–290. doi: 10.1002/9780470942390.mo140295, PMID: 26629772 PMC4686373

[ref12] Gomez-Ocadiz R. Silberberg G. (2023). Corticostriatal pathways for bilateral sensorimotor functions. Curr. Opin. Neurobiol. 83:102781. doi: 10.1016/j.conb.2023.102781, PMID: 37696188

[ref13] Grillner S. El Manira A. (2020). Current principles of motor control, with special reference to vertebrate locomotion. Physiol. Rev. 100, 271–320. doi: 10.1152/physrev.00015.2019, PMID: 31512990

[ref14] Haggard P. (2009). Neuroscience. The sources of human volition. Science 324, 731–733. doi: 10.1126/science.1173827, PMID: 19423807

[ref15] Libet B. Gleason C. A. Wright E. W. Pearl D. K. (1983). Time of conscious intention to act in relation to onset of cerebral activity (readiness-potential). The unconscious initiation of a freely voluntary act. Brain 106, 623–642. doi: 10.1093/brain/106.3.623, PMID: 6640273

[ref16] Manzanares G. Brito-da-Silva G. Gandra P. G. (2018). Voluntary wheel running: patterns and physiological effects in mice. Braz. J. Med. Biol. Res. 52:e7830. doi: 10.1590/1414-431X20187830, PMID: 30539969 PMC6301263

[ref17] Meijer J. H. Robbers Y. (2014). Wheel running in the wild. Proc. Biol. Sci. 281:20140210. doi: 10.1098/rspb.2014.0210, PMID: 24850923 PMC4046404

[ref18] Nicholas M. A. Yttri E. A. (2024). Motor cortex is responsible for motoric dynamics in striatum and the execution of both skilled and unskilled actions. Neuron 112, 3486–3501 e 3485. doi: 10.1016/j.neuron.2024.07.022, PMID: 39168128

[ref19] Orlovsky G. N. Deliagina T. G. Grillner S. (1999). Neural control of locomotion. Oxford, England: Oxford Univ. Press.

[ref20] Passingham R. E. Bengtsson S. L. Lau H. C. (2010). Medial frontal cortex: from self-generated action to reflection on one's own performance. Trends Cogn. Sci. 14, 16–21. doi: 10.1016/j.tics.2009.11.001, PMID: 19969501 PMC2806969

[ref21] Roseberry T. K. Lee A. M. Lalive A. L. Wilbrecht L. Bonci A. Kreitzer A. C. (2016). Cell-type-specific control of brainstem locomotor circuits by basal ganglia. Cell 164, 526–537. doi: 10.1016/j.cell.2015.12.037, PMID: 26824660 PMC4733247

[ref22] Schmitt A. Herzog P. Rochner F. Brandle A. L. Fragasso A. Munz B. (2020). Skeletal muscle effects of two different 10-week exercise regimens, voluntary wheel running, and forced treadmill running, in mice: a pilot study. Physiol. Rep. 8:e14609. doi: 10.14814/phy2.14609, PMID: 33118684 PMC7594150

[ref23] Schurger A. Hu P. Pak J. Roskies A. L. (2021). What is the readiness potential? Trends Cogn. Sci. 25, 558–570. doi: 10.1016/j.tics.2021.04.001, PMID: 33931306 PMC8192467

[ref24] Shepherd G. M. (2013). Corticostriatal connectivity and its role in disease. Nat. Rev. Neurosci. 14, 278–291. doi: 10.1038/nrn3469, PMID: 23511908 PMC4096337

[ref25] Tennant K. A. Adkins D. L. Donlan N. A. Asay A. L. Thomas N. Kleim J. A.. (2011). The organization of the forelimb representation of the C57BL/6 mouse motor cortex as defined by intracortical microstimulation and cytoarchitecture. Cereb. Cortex 21, 865–876. doi: 10.1093/cercor/bhq159, PMID: 20739477 PMC3059888

[ref26] Terstege D. J. Epp J. R. (2024). PAW, a cost-effective and open-source alternative to commercial rodent running wheels. Hardware X 17:e00499. doi: 10.1016/j.ohx.2023.e00499, PMID: 38204596 PMC10776975

[ref27] Triggiani A. I. Kreiman G. Lewis C. Maoz U. Mele A. Mudrik L.. (2023). What is the intention to move and when does it occur? Neurosci. Biobehav. Rev. 151:105199. doi: 10.1016/j.neubiorev.2023.105199, PMID: 37119992 PMC10330627

[ref28] Ueno M. Yamashita T. (2011). Kinematic analyses reveal impaired locomotion following injury of the motor cortex in mice. Exp. Neurol. 230, 280–290. doi: 10.1016/j.expneurol.2011.05.006, PMID: 21619878

[ref29] Xu T. Yu X. Perlik A. J. Tobin W. F. Zweig J. A. Tennant K.. (2009). Rapid formation and selective stabilization of synapses for enduring motor memories. Nature 462, 915–919. doi: 10.1038/nature08389, PMID: 19946267 PMC2844762

[ref30] Yang G. Pan F. Gan W. B. (2009). Stably maintained dendritic spines are associated with lifelong memories. Nature 462, 920–924. doi: 10.1038/nature08577, PMID: 19946265 PMC4724802

[ref31] Zorner B. Filli L. Starkey M. L. Gonzenbach R. Kasper H. Rothlisberger M.. (2010). Profiling locomotor recovery: comprehensive quantification of impairments after CNS damage in rodents. Nat. Methods 7, 701–708. doi: 10.1038/nmeth.1484, PMID: 20836253

