# Supplementary figures and images for: Volitional and forced running ability in mice lacking intact primary motor cortex
Source: Front Neural Circuits. 2025 Aug 14;19:1630932. doi: 10.3389/fncir.2025.1630932 (PMC12391011; doi:10.3389/fncir.2025.1630932)

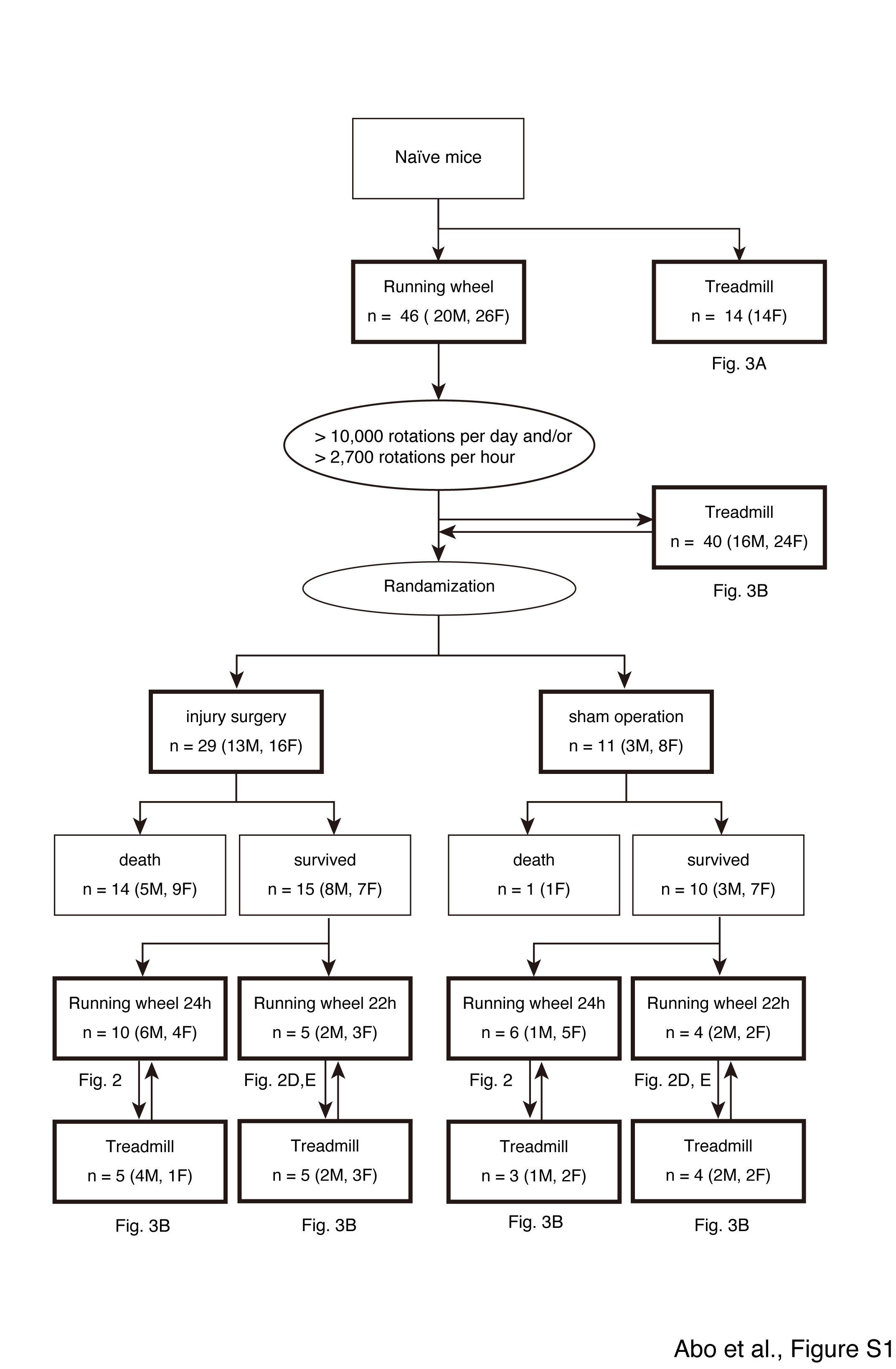

Supplement: Supplementary Figure S1 — Flowchart of the experiments with running wheels and treadmill. M: male, F: female. [file Image_1.tif]

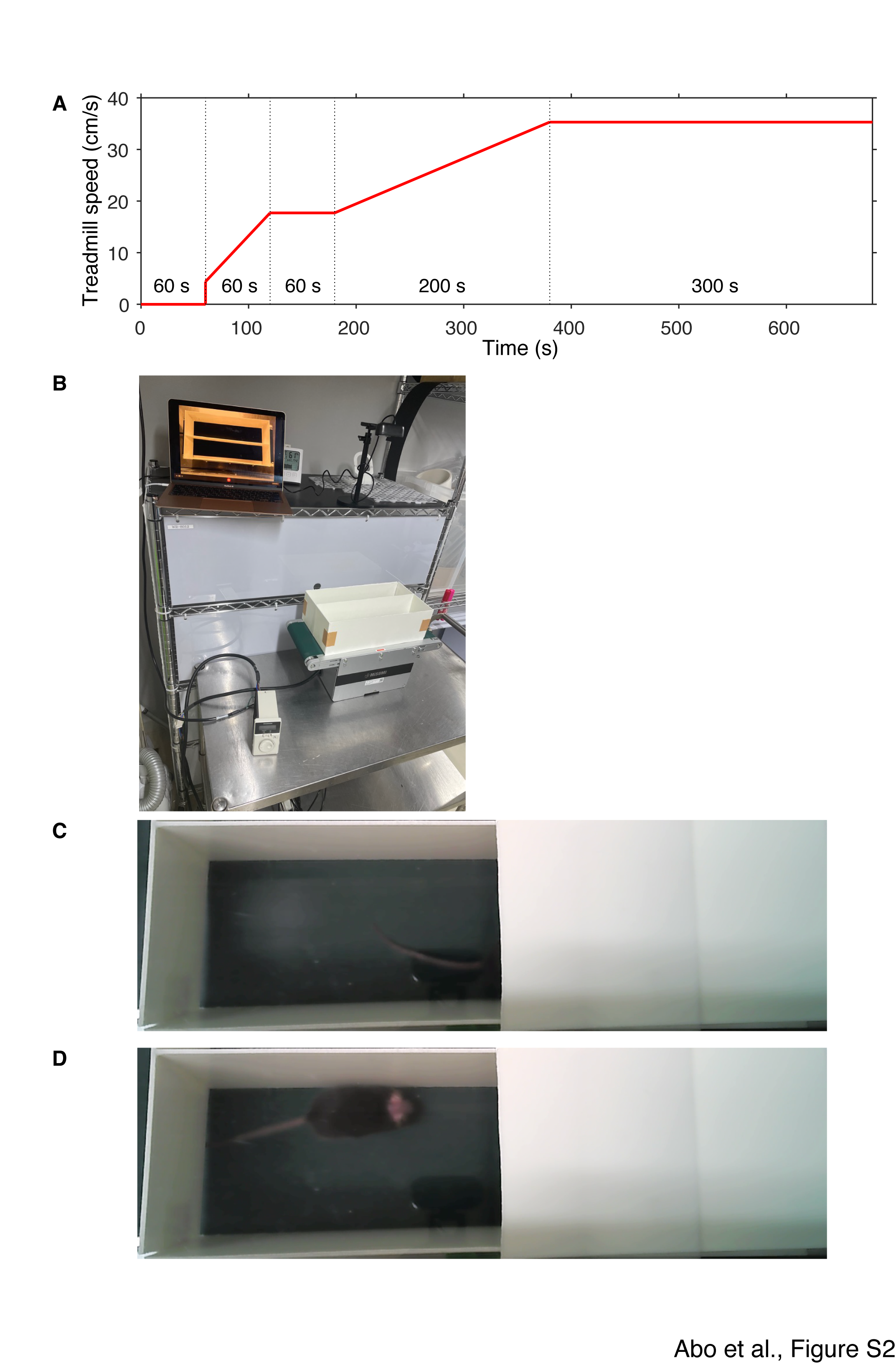

Supplement: Supplementary Figure S2 — Forced running experiment on a treadmill. (A) Speed of treadmill rotation. (B) Treadmill experimental apparatus. (C) Video image during running. (D) Video image of mice unable to keep up with the speed of the treadmill. [file Image_2.tif]

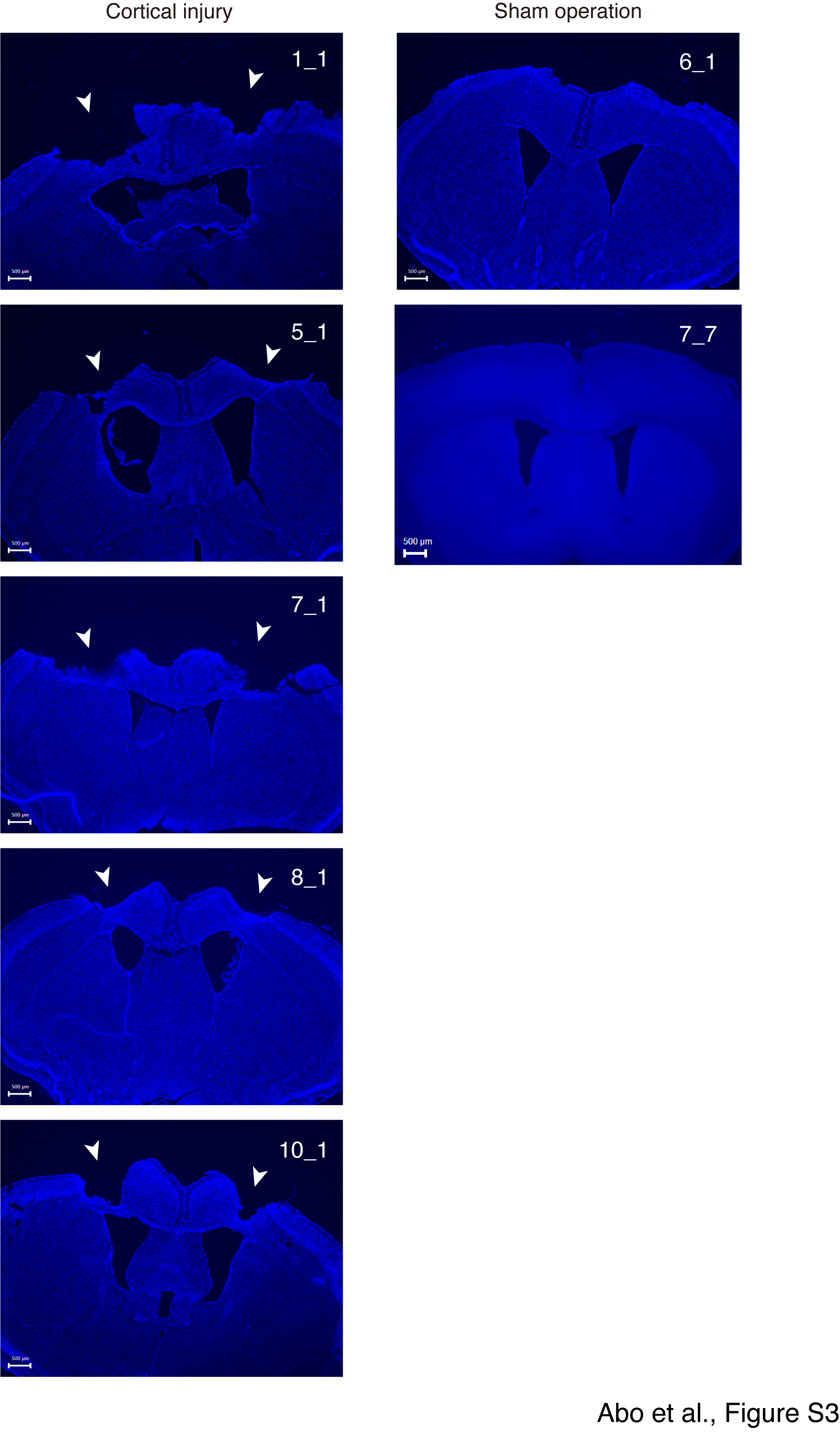

Supplement: Supplementary Figure S3 — DAPI staining images. The cerebral cortical sections of mice (1_1, 5_1, 7_1, 8_1, and 10_1) after 2, 3, 1, 4 and 4 weeks after cortical injury surgery, respectively, in the left, and the sections of mice (6_1, and 7_7) 2 weeks after sham-operation in the right. Injured sites are indicated by arrowheads. Scale bars: 500 μm. [file Image_3.tif]

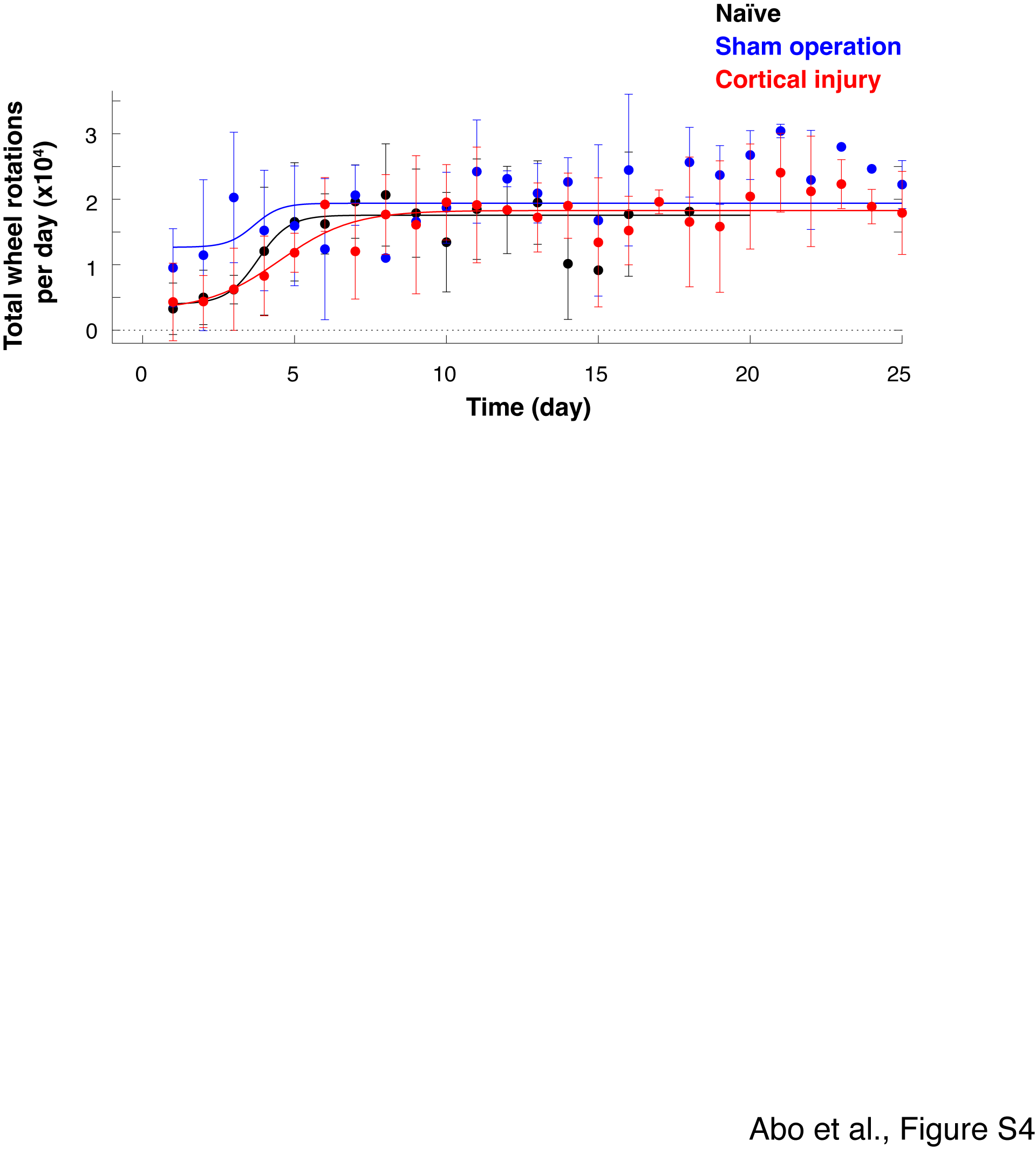

Supplement: Supplementary Figure S4 — Effects of cortical damage on volitional locomotion. Total wheel rotations per day before and after surgery plotted against elapsed days (mean ± standard deviation). The data shown in Figures 2A–C are superimposed on a single figure. [file Image_4.tif]
